# Supplementary material for: Colossal thermo-hydro-electrochemical voltage generation for self-sustainable operation of electronics
Source: Nat Commun. 2021 Sep 6;12:5269. doi: 10.1038/s41467-021-25606-3 (PMC8421453; doi:10.1038/s41467-021-25606-3)
Supplement: Supplementary file 1 — Supplementary Information [file 41467_2021_25606_MOESM1_ESM.pdf]

SUPPLEMENTARY INFORMATION

Colossal Thermo-Hydro-Electrochemical Voltage Generation for

Self-sustainable Operation of Electronics

Yufan Zhang[1], Ahrum Sohn[2], Anirban Chakraborty[2], Choongho Yu[1,2] *

[1] Department of Materials Science and Engineering, Texas A&M University, College Station, Texas, 77843 USA

[2] Department of Mechanical Engineering, Texas A&M University, College Station, Texas, 77843 USA

*Corresponding author: chyu@tamu.edu

1. Material Characterization

PANI:PSS was characterized by attenuated total reflectance (ATR) Fourier transform infrared spectroscopy (FTIR) (Thermo Nicolet 380 FTIR spectrometer), as shown in Supplementary Fig. 1. The peaks at 1006 cm^{-1} and 1128 cm^{-1} correspond to benzene in-plane bending vibrations and skeleton vibrations, respectively. Out-of-plane deformation of C-H in the benzene ring is in the region of 823 cm^{-1}. Symmetric-stretching vibration of the sulfonic group attributes to the peak at 1034 cm^{-1}. The C-N stretching of the secondary aromatic amine appears at 1303 cm^{-1}. C-C stretching deformation of quinoid and C-C stretching deformation of benzenoid rings are at 1606 m^{-1} and 1490 cm^{-1}, respectively. These peaks match those in the literature [1,2], which indicates that our PANI:PSS was synthesized successfully. The surface of electrodes was inspected by a JEOL JSM-7500F field emission scanning electron microscope. X-ray diffraction patterns were obtained using a Bruker D8 Discover X-ray diffractometer. Electrochemical impedance spectra was obtained by a Gamry Interface 1010 potentiostat, and Tafel curves were obtained using a CHI 604D electrochemical analyzer. The thermal conductivity of our whole device along the out-of-plane direction was measured to be 0.41 W m^{-1} K^{-1} using the ASTM D5470 method.

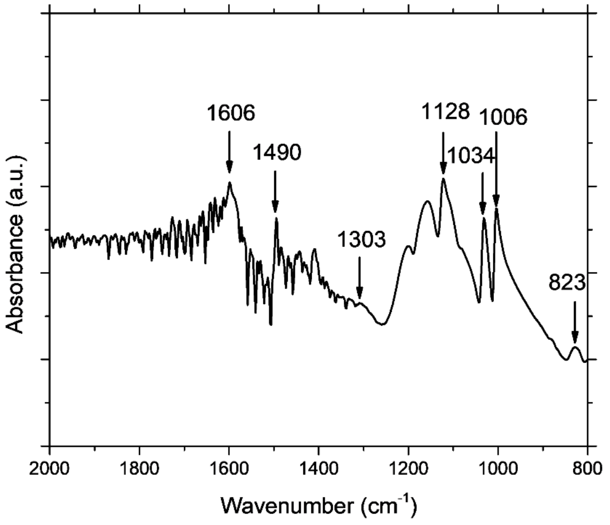

Supplementary Fig. 1. ATR-FTIR of PANI:PSS.

2. Water Uptake Measurement

Initially PANI:PSS was kept in an ambient condition (~22 ℃) with 50 % relative humidity (RH). Then, PANI:PSS was placed in a humidity-controlled chamber, as described in our earlier work [3]. The mass of PANI:PSS was recorded every two hours. The water uptake after 8 h was saturated and plotted in Fig. 1f. Thermal-to-electrical conversion factor (TtoE factor) measurements were carried out after samples were left under the controlled humidity environment for 8 h. The water uptake was calculate as follows.

$$\text{Water uptake (\%)} = \frac{m_1 - m_{FD}}{m_{FD}} \times 100 \quad (1)$$

where m_1 is the total mass of PANI:PSS after it was exposed to an environment with different relative humidity. m_{FD} is the mass of fully dried PANI:PSS by evaporating water in a vacuum oven at 50 ℃ for 48 h.

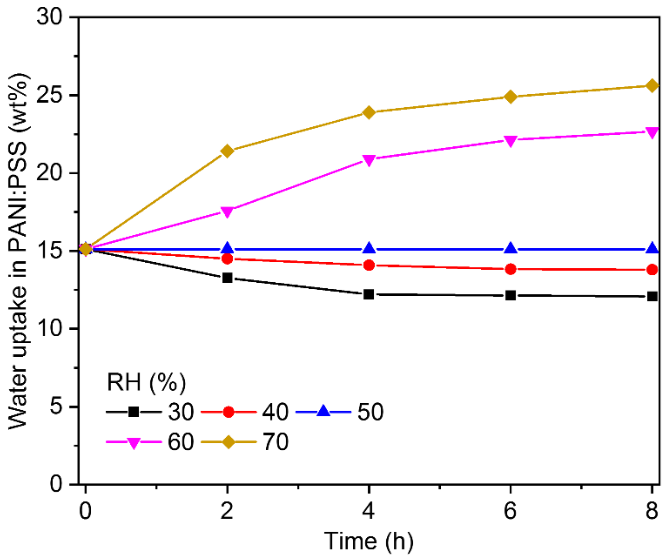

Supplementary Fig. 2. Water uptake in PANI:PSS as a function of time under 30%, 40%, 50%, 60% and 70% RH.

3. TtoE factor Measurement

TtoE factors were measured using our custom-built setup (See Supplementary Fig. 3a), which is also described in our previous work [3-5].

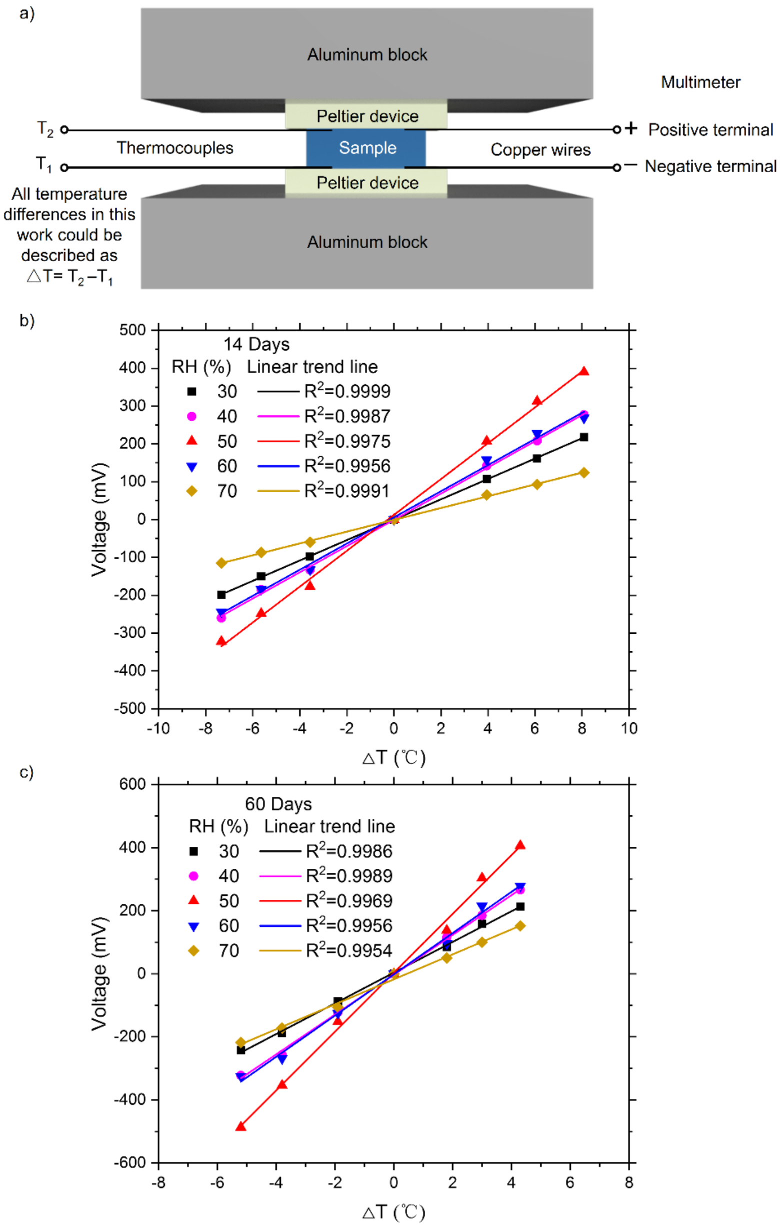

Supplementary Fig. 3. (a) Experimental setup for TtoE factor measurements. Voltage *vs.* temperature difference under different relative humidity (RH) for devices with oxidation layers after (b) 14 days and (c) 60 days.

Supplementary Table 1. Slope and R^2 for the linear fit in Supplementary Fig. 3b.

Relative humidity (% RH)	Slope (mV K^{-1})	R^2
30	27	0.9999
40	35	0.9987
50	47	0.9975
60	35	0.9956
70	16	0.9991

Supplementary Table 2. Slope and R^2 for the linear fit in Supplementary Fig. 3c.

Relative humidity (% RH)	Slope (mV K^{-1})	R^2
30	48	0.9985
40	63	0.9987
50	93	0.9962
60	65	0.9947
70	39	0.9945

4. Electrochemical Impedance Spectroscopy (EIS) Measurement

Nyquist plots are displayed in Supplementary Fig. 4a, 4b, 4c, 4d and 4e. The impedance of the device decreased under higher relative humidity whereas the impedance increased under lower relative humidity. This implies that water uptake strongly affects the charge transfer. The semicircles indicating capacitive behaviors can be attributed to the electrically insulating FeOOH layer on carbon steel electrodes. Larger semicircles were observed from the device with the oxidation layer after 60 days than that of 14 days. This indicates that the impedance was increased due to more oxidation layers between the carbon steel electrodes and PANI:PSS. It should be noted that the oxidation layer was no longer developed further afterwards.

When graphite foil electrodes were used instead of carbon steel electrodes without the FeOOH layer, the impedance was significantly lowered, as depicted in Supplementary Fig. 4f. We believe that the electrically insulating FeOOH layers plays an important role in creating large potential differences between two electrodes. The pores in FeOOH layer facilitates a large swing in water uptake, differentiating the potential of the two electrodes depending on the water uptake. Conversely, the graphite electrodes without the FeOOH layer is unfavorable to maintain a potential difference, if any, due to the relatively low impedance between the two graphite electrodes.

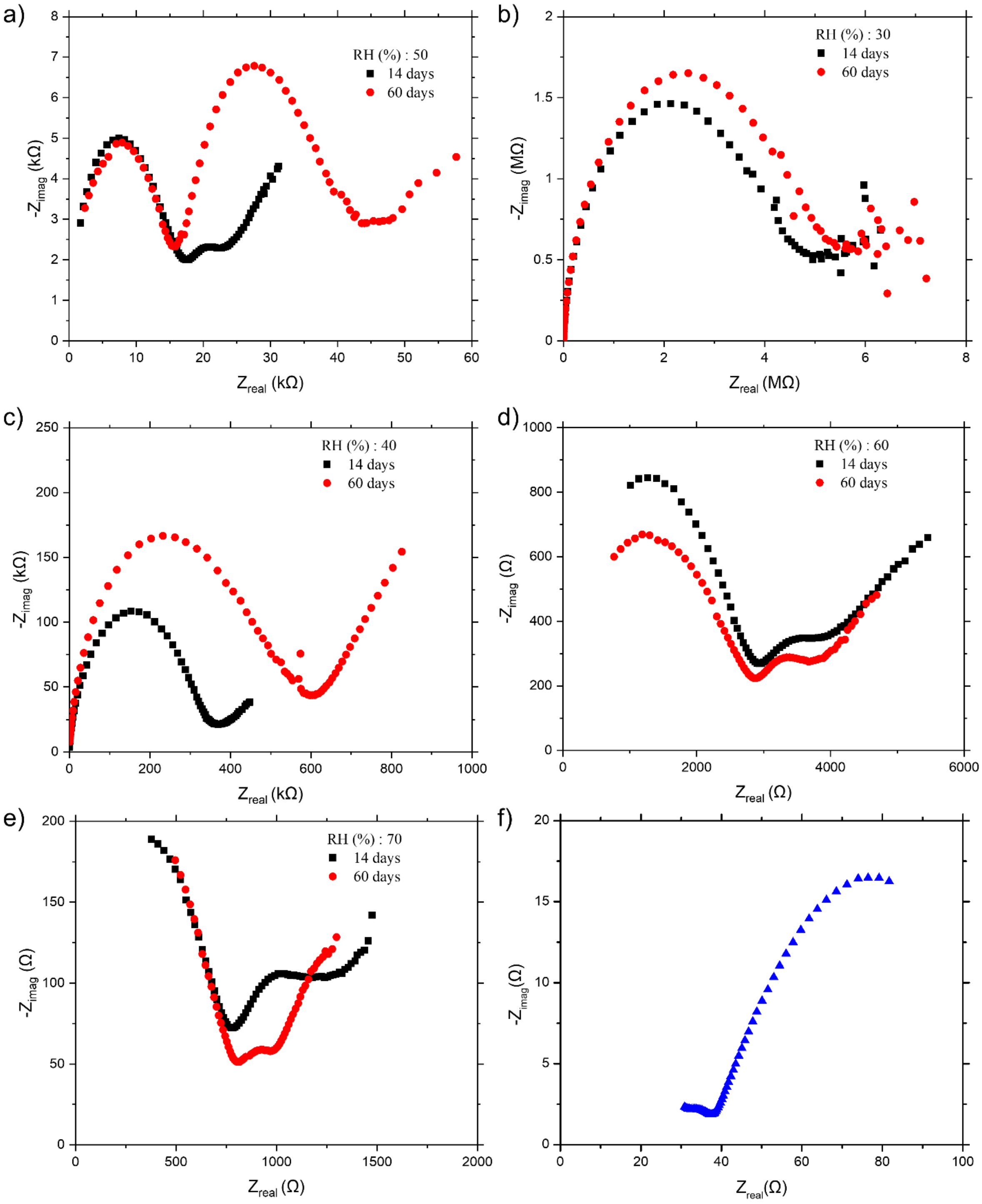

Supplementary Fig. 4. (a) Nyquist plots from electrochemical impedance spectroscopy measurement of the device shown in Fig. 1 under (a) 50; (b) 30; (c) 40; (d) 60; (e)70 %RH. (f) Nyquist plot of a test sample made of PANI:PSS and graphite electrodes instead of carbon steel electrodes.

5. Investigation of Working Mechanism

We have carried out systematic studies to unveil the working principle of our device. Rigorous literature survey and experiments brought us five different possible hypotheses as major causes for the voltage generation under temperature difference.

5.1. Ion concentration difference between the hotter and colder sides

The thermo-diffusion of ions in polyelectrolytes under a temperature gradient makes the ion concentration in the hotter and colder side different. Kim *et al.* described the relationship between proton concentration and voltage in PSS-H using the Nernst equation: [3]

$$E_{cell} = E_{cell}^{0} - \frac{RT}{F} ln \frac{[H_{hot}^{+}]}{[H_{cold}^{+}]} \quad (2)$$

where E_{cell} is the voltage between hotter side and colder side; E_{cell}^{0} is the standard cell potential from two electrodes; R is the universal gas constant; T is the temperature; F is the Faraday constant; and $[H_{hot}^{+}]$ and $[H_{cold}^{+}]$ are the proton concentration in the hotter side and colder side.

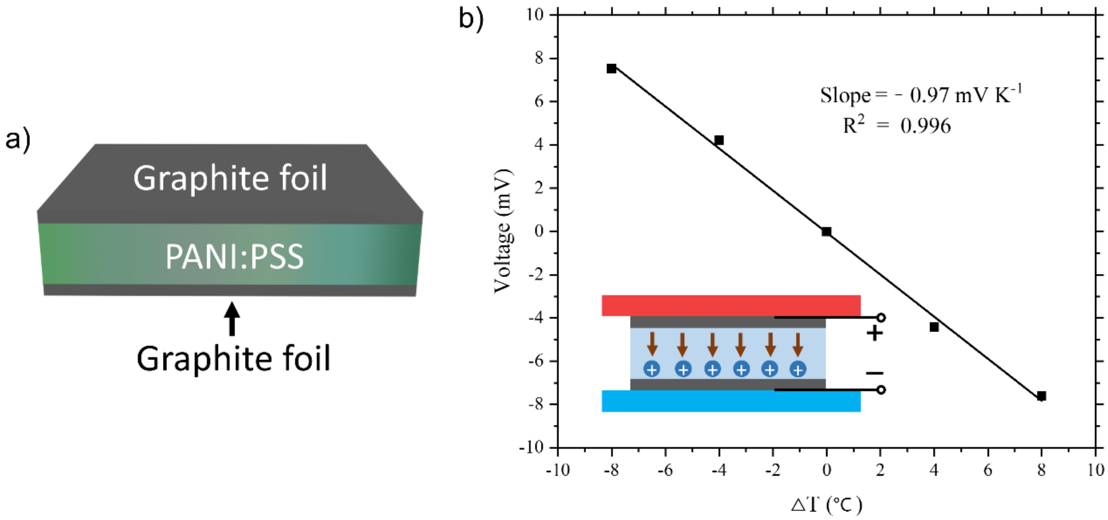

Supplementary Fig. 5. (a) Device configuration of PANI:PSS as electrolyte and graphite foils as electrodes. (b) Voltage *vs.* temperature difference plot with the trend line for measuring TtoE factor. The slope is negative, so the TtoE factor is positive like conventional thermpower.

To investigate the influence of the ion concentration difference on the TtoE factor, carbon steel electrodes were substituted by graphite foils so as to eliminate the corrosion effect from carbon steel, as illustrated in Supplementary Fig. 5a. Based on the relationship between temperature difference and voltage under the ambient condition, a negative slope of –0.97 mV K^{-1} from the linear fitting was obtained. When a temperature difference is created, protons migrates from the hotter to the colder side, as illustrated in the inset of Supplementary Fig. 5b. Then the colder side has a higher potential due to a higher concentration of positive ions, resulting in a negative slope. This trend is opposite to that of our PANI:PSS (see the inset of Fig. 2a). Furthermore, the absolute value of the slope is much smaller than the TtoE factor of our device. Therefore, we concluded that the thermo-diffusion of ions is not the decisive contributor of the TtoE factor observed in our carbon steel based device shown in Fig. 1a.

5.2. Comparative study about the working principles of our devices and moisture-powered devices

Moisture-powered devices generate voltage by carrying ions (protons) during water diffusion through a hygroscopic medium and thereby inducing charge imbalance when one of the two sides in the medium is exposed to more water [6,7]. In literature, metal and carbon electrodes (e.g., gold, graphite) were used together with a hygroscopic materials in between [7,8]. In our work, we assembled two devices with carbon steel or graphite foil electrodes in Supplementary Fig. 6a or 6b, respectively. We sealed the side walls with silicone to ensure that water pass through PANI:PSS without evaporation from the side walls. When the devices initially at a 27% RH environment were moved to a 85% RH environment, the voltage from the device with carbon steel electrodes was changed to a large negative voltage, –360 mV (See Supplementary Fig. 6c) whereas

that of the graphite foil electrodes displayed less than +1 mV (See Supplementary Fig. 6d). This result shows that ion migration due to water diffusion is not a major contributor to the voltage generation in our devices unlike the moisture-powered devices. Instead, more water near the hole side gave rise to more negative potential than the other side.

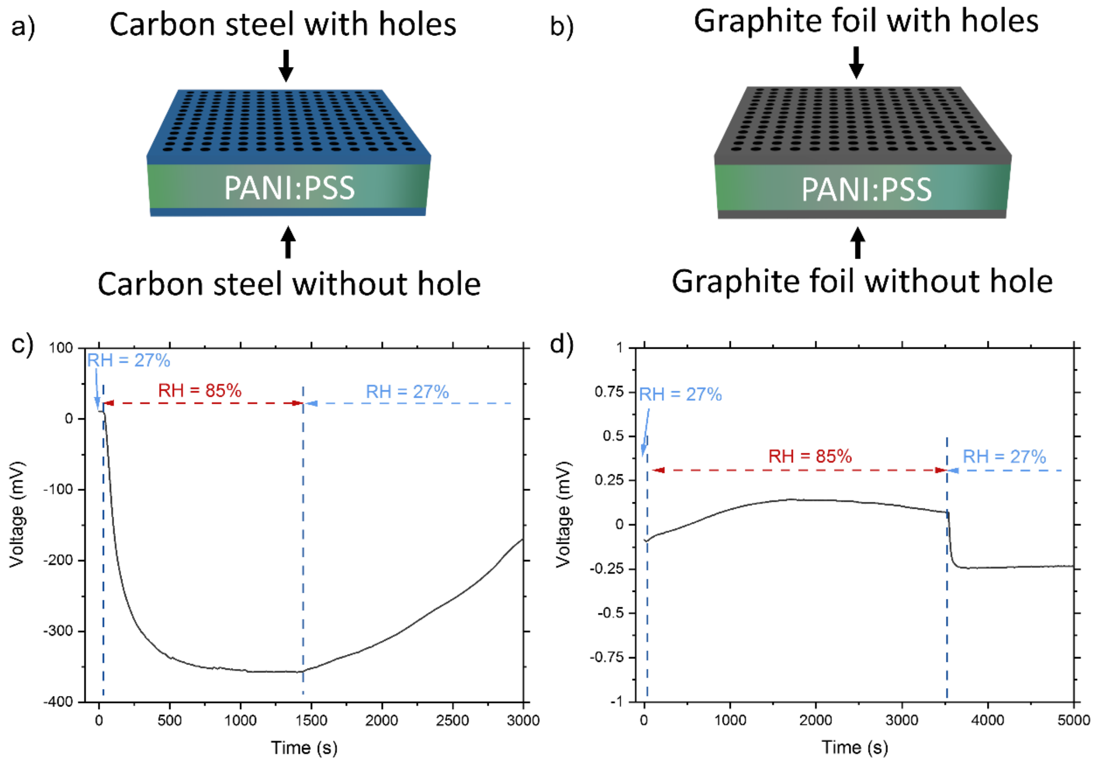

Supplementary Fig. 6. (a) Device configuration of PANI:PSS as an electrolyte and carbon steels with holes and without holes as electrodes. (b) Device configuration of PANI:PSS as an electrolyte and graphite foils with holes and without hole as electrodes. Voltage response as a function of time under humidity change in the devices with (c) carbon steel electrodes and (d) graphite electrodes.

5.3. Difference in the redox potential between the hotter and colder sides

We also tested if the thermoelectric voltage in our device can be attributed to the redox reaction of $FeOOH/Fe^{2+}$, which is described as follows.

$$FeOOH + 3H^{+} + e^{-} \rightleftharpoons Fe^{2+} + 2H_2O \quad (3)$$

We designed an experiment using FeOOH products from the carbon steel electrode of our device in a configuration depicted in Supplementary Fig. 7a. To attach the FeOOH products to the graphite foils, the FeOOH products were spread on the graphite foils and two drops of a liquid-type superglue were applied and subsequently more FeOOH products were spread on top of the first layer. Then PANI:PSS was sandwiched between the two graphite electrodes. In this configuration, FeOOH and Fe^{2+} are provided by corrosive products and H^+ and H_2O are from polyelectrolyte. To investigate the temperature effect of this redox reaction, voltage change was recorded when temperature gradients were created. Supplementary Fig. 7b displays varying voltage responses at a constant temperature difference with small thermoelectric voltage. This result indicates that temperature-dependent redox reactions from FeOOH/Fe^{2+} are not the major cause for the colossal TtoE factor observed in our carbon steel based device shown in Fig. 1a.

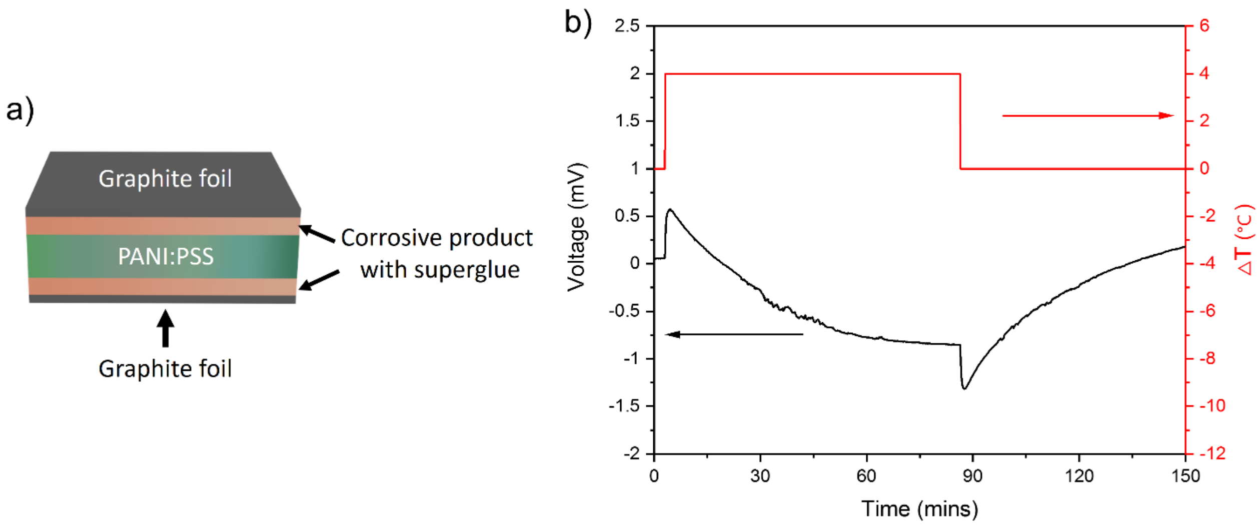

Supplementary Fig. 7. (a) A testing device configured for temperature-dependent redox reactions. Corrosive FeOOH products were sandwiched between a graphite foil electrode and PANI:PSS. (b) Voltage response as a function of time under a temperature difference.

5.4. Variation of corrosion potential due to the change in the ion concentration

When protons diffuse under a temperature gradient, it is possible to change the corrosion potential like pH-dependent corrosion of carbon steel [9,10]. Here, we designed an experiment to find out the relationship between pH and the corrosion potential of carbon steel. Tafel curve is one of the most effective method to measure the corrosion potential [11].

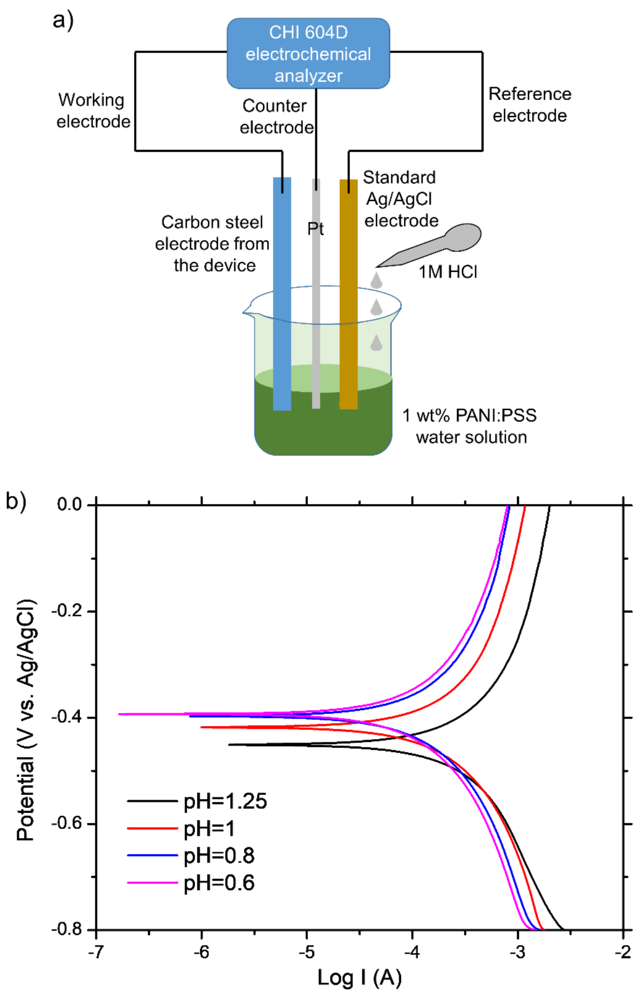

Supplementary Fig. 8. (a) Three electrodes configuration for the measurement of the Tafel curve while 1M HCl was added to the solution. (b) Tafel curves for the carbon steel electrode taken out of our device in the solutions at different pH.

Supplementary Fig. 8a depicts our experimental setup consisting of a working electrode from our device (shown in Fig. 1), Pt counter electrode, and Ag/AgCl reference electrode in an aqueous 1 wt% PANI:PSS solution. Initially, pH was measured to be 1.25. Tafel curves were recorded as 1M HCl was added to have pH=1, 0.8, and 0.6, as displayed in Supplementary Fig. 8b. When the current between the working and counter electrode became small, the corrosion potential between the working and reference electrodes was changed from -0.45 V to -0.39 V with diminishing pH (*i.e.*, increasing proton concentration). The potential gets higher with more protons, which is similar to the inset of Supplementary Fig. 5b. This denotes our device in the main manuscript operates under a different working principle.

We designed another experiment with 1M $FeCl_3$ solution as an electrolyte and the carbon steel electrodes detached from the device as electrodes, as illustrated in Supplementary Fig. 9a since Fe ions participate in the corrosion process. Voltage was measured as concentrated HCl (12M) was added to the solution where the positive terminal was immersed. Supplementary Fig. 9b displays positively increasing voltage with more protons, denoting that the potential is higher with more protons like the trends shown in Supplementary Fig. 8 and Supplementary Fig. 5. Therefore, we excluded this as a major contributor for the TtoE factor in our device.

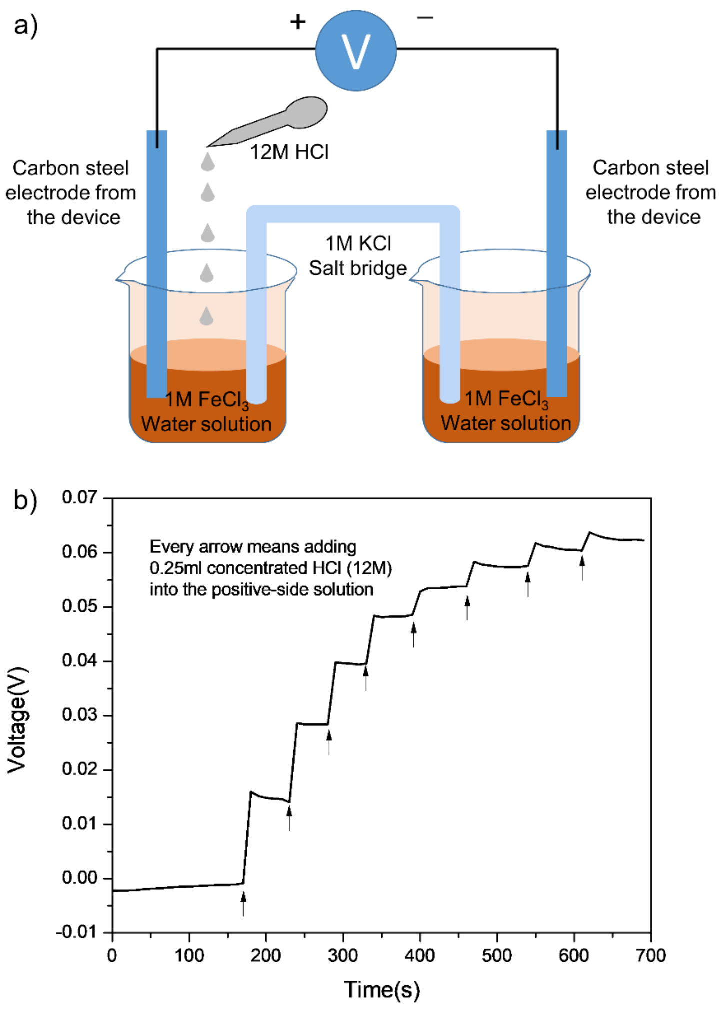

Supplementary Fig. 9. (a) Experimental setup for measuring voltage as HCl was gradually added. (b) Voltage profile as a function of time when HCl was added to the solution where the carbon steel electrode connected to the positive terminal was immersed.

5.5. Corrosion potential due to the change in temperature

The experimental setup in Supplementary Fig. 8a was used again, but at this time the temperature of the entire beaker was immersed in a water bath instead of HCl addition, as illustrated in Supplementary Fig. 10a. The water bath was used for facilitating uniform temperature of the PANI:PSS solution during the experiment. The corrosion potentials of the electrode at different temperatures are displayed in the Tafel curve (Fig. 10b).

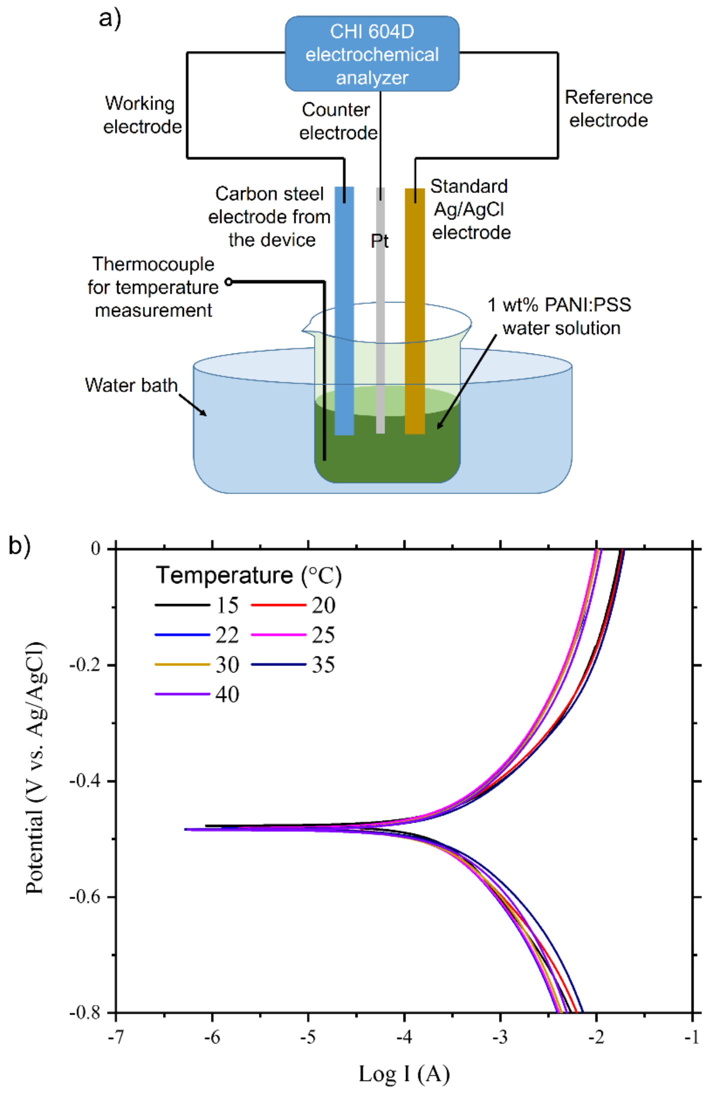

Supplementary Fig. 10. (a) Three electrode configuration for the measurement of the Tafel curves at different temperatures. (b) Tafel curves for the carbon steel electrode detached from our device when the solution temperature was varied.

We observed only 7 mV difference while the temperature changed from 15 ℃ to 40 ℃. When the temperature was raised, the corrosion potential was lowered. This trend is also different from our device. Temperature effect on the corrosion potential is much smaller than the colossal TtoE factor from our device, suggesting temperature variation is also not the major driver of our system.

5.6. Corrosion potential due to the change of water mass in the electrodes

Carbon steel corrodes when an electrochemical cell is formed on its surface. Supplementary Fig. 11a shows a cross-section of a carbon steel electrode after 6 months. The thickness of the carbon steel has been reduced from ~125 μm to ~107 μm after 6 months. The average reduction in thickness assuming a constant corrosion rate (which is in fact not constant) is only ~3 μm $month^{-1}$. Four essential elements for this electrochemical cell for corrosion include (a) anodic reaction, (b) cathodic reaction, (c) metallic path between anodic and cathodic sites, and (d) electrolyte, as illustrated in Supplementary Fig. 11b. Anodic and cathodic sites are in carbon steel surface, and iron metal connects these two sites so that electrons can pass through. Water is the most common electrolyte for carbon steel corrosion. Yan et al. studied how the amount of water in a soil environment (carbon steel buried in soil) affects carbon steel corrosion [12]. This study delineated that water uptake less than 30% did not cover all the surfaces of the carbon steel. Then electrochemical cells on the surface of the carbon steel would form in the area where water was present. Water is the electrolyte so corrosion is strongly dependent on the amount of water at the local site, which can be described by the overpotential ($E_{overpotential} = I_iR$, where I_i is the corrosion current and R_i is the resistance of electrolyte). With more water, the resistance becomes smaller so the overpotential becomes smaller and vice versa, as described in the main manuscript along with Fig. 3d. Water in PANI:PSS diffuses upon imposing a temperature gradient. Then, the amounts of water in the two carbon steel electrodes become different, resulting in dissimilar overpotentials, and thereby nonzero voltage between the two electrodes are observed.

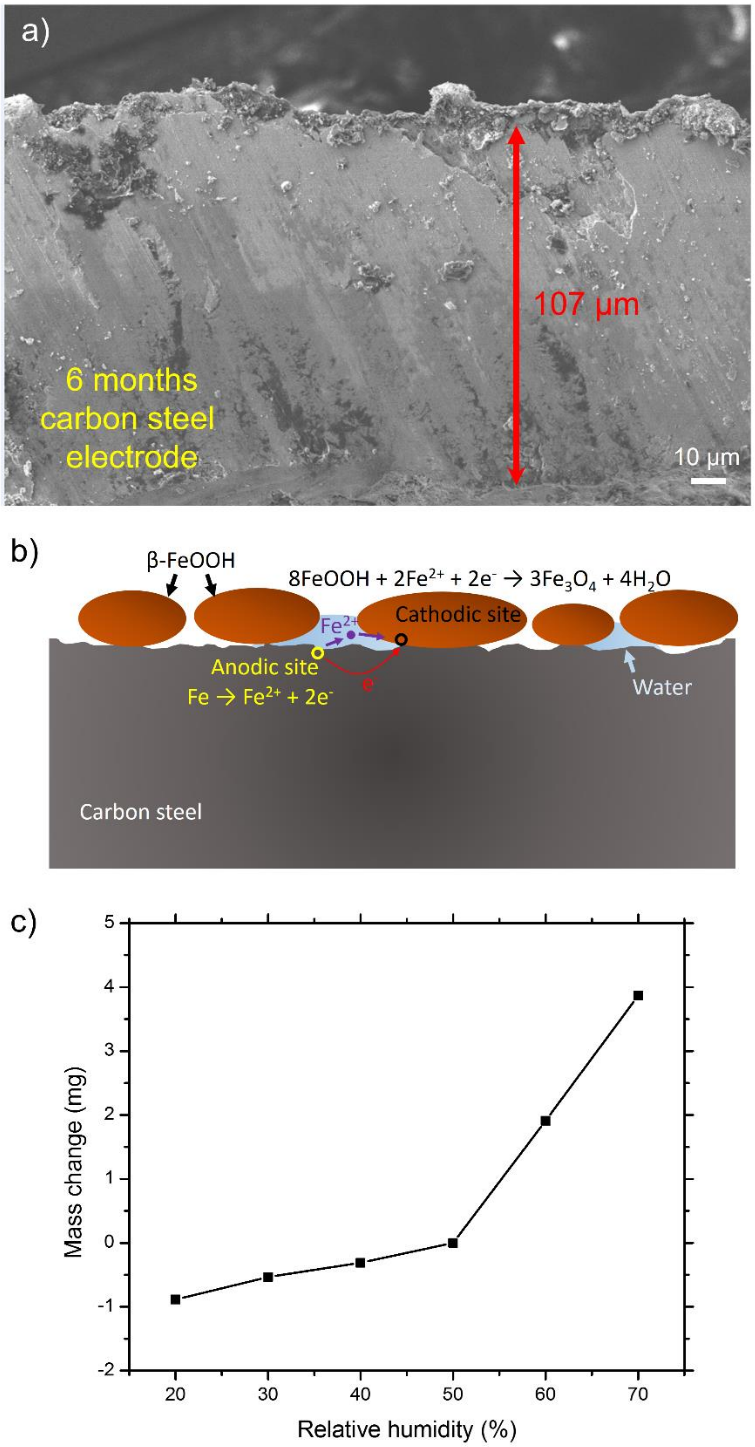

Supplementary Fig. 11. (a) SEM image of a carbon steel electrode (cross section) after 6 months. The thickness of the electrode was reduced to ~107 μm from the initial thickness of ~125 μm, showing ~3 μm month^{-1} reduction in thickness. (b) Illustration of the carbon steel electrode where FeOOH covered its surface with water in between. (c) Mass change in a single carbon steel electrode after the sample was left under different relative humidity for 12 h.

6. In-situ ATR-FTIR for Investigating Thermo-diffusion of Water

In-situ ATR-FTIR was carried out with a configuration in the inset of Fig. 3c. PANI:PSS was placed on the crystal of ATR accessory and a heater was placed on top of the PANI:PSS sample. The heater was pressed from the top to ensure PANI:PSS makes good contacts with the heater and the crystal surface. The first scan was carried out at room temperature in the range of 4000~2000 cm^{-1} without turning on the heater. Subsequently, the heater was turned on. After about 30 seconds, the scanning began and, after about 90 seconds, it was finished. The thermocouples recorded the temperature (T_1 and T_2) at the end of the scan. Then the power of the heater was raised, and the ATR-FTIR and temperature data were recorded. This procedure was repeated 4 times to obtain the FTIR absorbance spectra as a function of temperature difference. Supplementary Table 3 includes temperature measurement results.

Supplementary Table 3. Temperature during the in-situ ATR-FTIR measurement.

Time (min)	T_2 (℃)	T_1 (℃)	Temperature difference (℃)
0	23.6	23.6	0.0
2	25.8	24.3	1.5
4	27.6	24.5	3.1
6	29.8	24.8	5.0
8	32.5	25.0	7.5

The peak at 2914 cm^{-1} corresponding to C-H stretching in CH_2 group of PSS was used to normalize FTIR spectra for better comparison. The O-H stretching band from water is broad over around 2800~3700 cm^{-1}, the intensity of nearby peaks at 3340 and 3240 cm^{-1}, corresponding to N-H stretching, could be affected by the change of the O-H peak.

7. Operation of Electrochromic Devices

Supplementary Fig. 12a shows an electrochromic device (Ynvisible Interactive Inc.) without any electrical connection. According to the specification, this electrochromic device shows patterns when electricity is supplied with a voltage of ~1.5 V. When the electrochromic device was hooked up with our serially connected four devices under a temperature difference of 5 ℃, the pattern has been changed (See Supplementary Fig. 12b), indicating that electricity was supplied to the electrochromic device. When the positive and negative terminals were switched, the pattern was reversed (See Supplementary Fig. 12c).

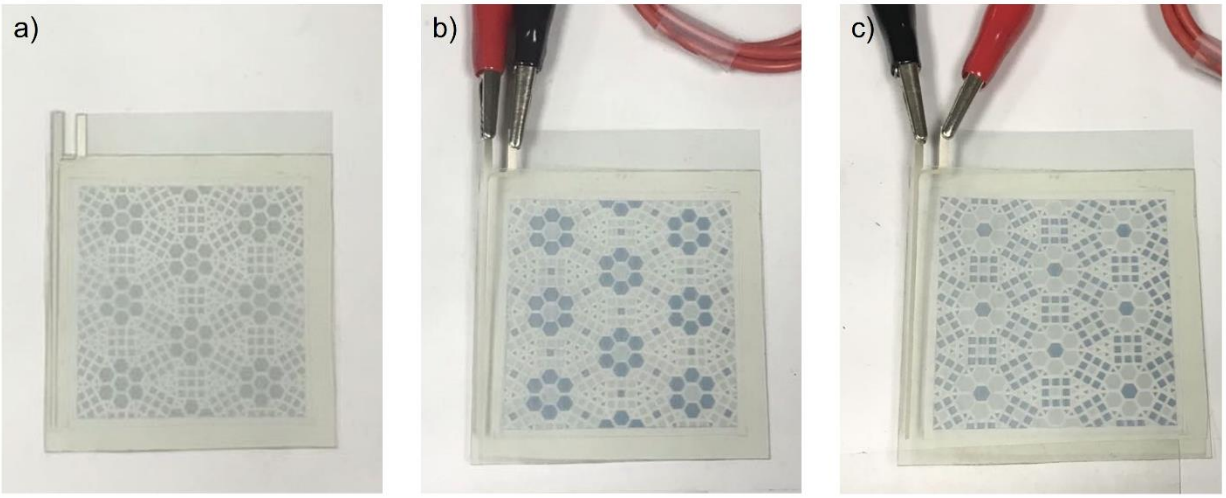

Supplementary Fig. 12. Photographs of an electrochromic device (a) without any electrical connection, (b) after connecting our devices (four in series) under a temperature difference of 5 ℃, and (c) after flipping the positive and negative terminals.

8. Self-sustainable Fever Detection

We have fabricated a fever indicator based on electrochromic color changes by following our earlier work [5]. The structure of our fever indicator is shown in Supplementary Fig. 13a. First, 0.0052-M HCl, 0.01-M $K_3Fe(CN)_6$ and 0.01-M $FeCl_3$ solution in DI water was prepared. A

prussian blue (PB) film was deposited on a ITO glass by a cyclic voltammetry method from 0 to 2 V with 0.05 V s^{-1} scan rate for 3 cycles in a 50-mL solution. We used a tape-masked ITO glass as the working electrode, Ag/AgCl as the reference electrode, and Pt as the counter electrode. Then, the PB film on the ITO glass was placed in a closed chamber where hydrazine was vaporized for 3 mins in order to reduce PB to prussian white (PW). To fabricate the electrochromic device, a Nafion 115 membrane was pre-soaked in a 0.125-M TEAP solution and then sandwiched between the ITO glass with PW and another bare ITO glass. Firnally, epoxy was used to seal the device to avoid the evaporation of the TEAP electrolyte.

We have estimated operating conditions when our energy harvesting device was used for those who have a fever. The heat dissipation from the humans without fever was found to be 200–500 W m^{-2} [13], so we assumed 360 W m^{-2} is a normal condition. In general, a body temperature above 38.1 ℃ is considered to be a fever [14]. It has been reported that ~13% increase in metabolism per 1 ℃ rise in the temperature of a human body [15,16]. Considering the maximum heat flux from healthy humans is 500 W m^{-2}, we assumed that the heat flux from humans with fever is 580 W m^{-2}. These values could be adjusted, if necessary, depending on needs and other considerations.

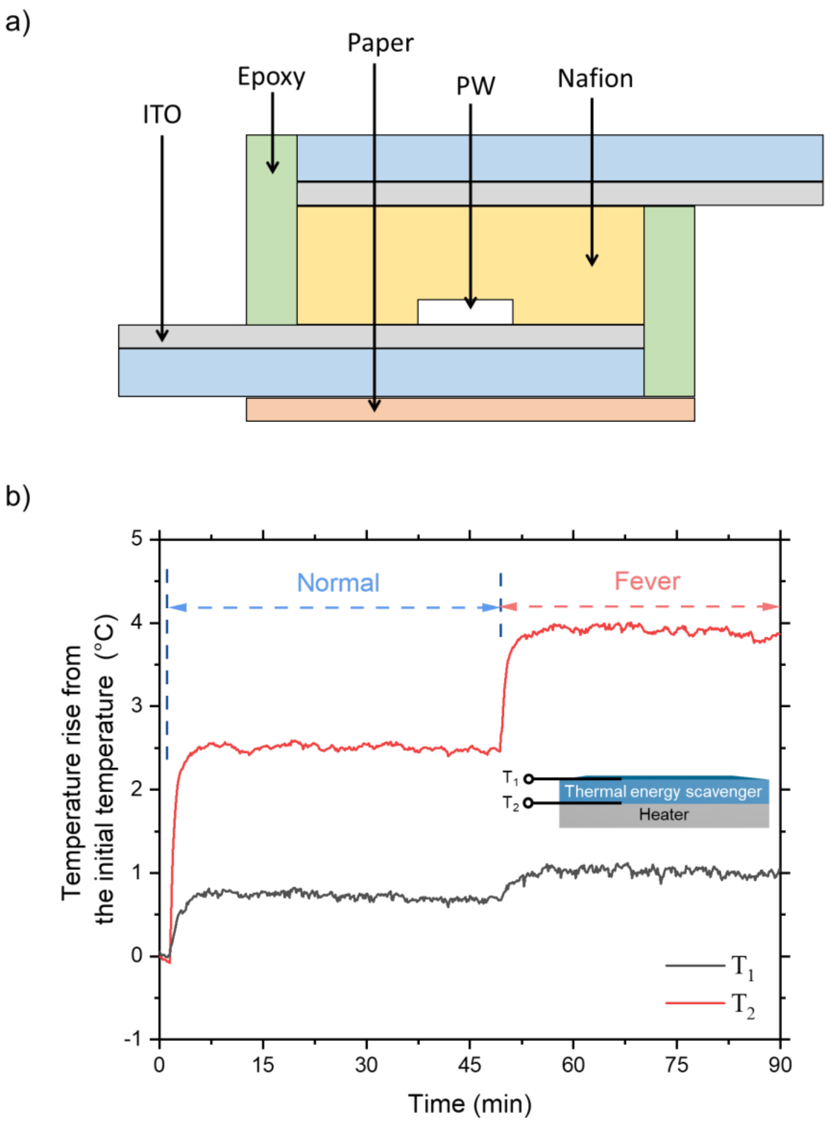

Supplementary Fig. 13. (a) Schematic illustration of the fever indicator. (b) Temperature rise of T_1 and T_2 from the initial temperature in our energy harvesting device as a function of time under two different heat flux conditions.

References

1 Li, Y., Ying, B., Hong, L. & Yang, M. Water-soluble polyaniline and its composite with poly (vinyl alcohol) for humidity sensing. *Synth Met* **160**, 455-461 (2010).
2 Stan, C. S., Popa, M., Olariu, M. & Secula, M. S. Synthesis and characterization of PSSA-polyaniline composite with an enhanced processability in thin films. *Open Chem.* **13**, 467-476, (2015).
3 Kim, S. L., Lin, H. T. & Yu, C. Thermally chargeable solid-state supercapacitor. *Adv. Energy Mater.* **6**, 1600546, (2016).
4 Kim, S. L., Hsu, J.-H. & Yu, C. Thermoelectric effects in solid-state polyelectrolytes. *Org. Electron.* **54**, 231-236, (2018).
5 Kim, S. L., Hsu, J.-H. & Yu, C. Intercalated graphene oxide for flexible and practically large thermoelectric voltage generation and simultaneous energy storage. *Nano Energy* **48**, 582-589, (2018).
6 Zhao, F., Wang, L., Zhao, Y., Qu, L. & Dai, L. Graphene Oxide Nanoribbon Assembly toward Moisture-Powered Information Storage. *Adv. Mater.* **29**, 1604972, (2017).
7 Xu, T. *et al.* An efficient polymer moist-electric generator. *Energy Environ. Sci.* **12**, 972-978, (2019).
8 Liang, Y. *et al.* Electric power generation via asymmetric moisturizing of graphene oxide for flexible, printable and portable electronics. *Energy Environ. Sci.* **11**, 1730-1735, (2018).
9 Pessu, F., Barker, R. & Neville, A. The influence of pH on localized corrosion behavior of X65 carbon steel in CO_2-saturated brines. *Corrosion* **71**, 1452-1466, (2015).
10 Zheng, Y., Ning, J., Brown, B. & Nešić, S. Electrochemical model of mild steel corrosion in a mixed H_2S/CO_2 aqueous environment in the absence of protective corrosion product layers. *Corrosion* **71**, 316-325, (2014).
11 McCafferty, E. *Introduction to Corrosion Science*. (Springer New York, 2010).
12 Yan, M., Sun, C., Xu, J. & Ke, W. Anoxic corrosion behavior of pipeline steel in acidic soils. *Ind Eng Chem Res* **53**, 17615-17624 (2014).
13 de Rivera, P. J. R., de Rivera, M. R., Socorro, F. & de Rivera, M. R. Measurement of human body surface heat flux using a calorimetric sensor. *J. Therm. Biol.* **81**, 178-184, (2019).
14 Herzog, L. W. & Coyne, L. J. What is fever? Normal temperature in infants less than 3 months old. *Clin Pediatr* **32**, 142-146 (1993).
15 Stettler, N., Schutz, Y., Whitehead, R. & Jéquier, E. Effect of malaria and fever on energy metabolism in Gambian children. *Pediatr. Res.* **31**, 102-106, (1992).
16 Du Bois, E. F. The mechanism of heat loss and temperature regulation. *Ann. Intern. Med.* **12**, 338-395, (1938).
